# Facile production of chlorophyllides using recombinant CrCLH1 and their cytotoxicity towards multidrug resistant breast cancer cell lines

**DOI:** 10.1371/journal.pone.0250565

**Published:** 2021-04-30

**Authors:** Yi-Ping Hsiang, Yi-Ting Wang, Keng-Shiang Huang, Ting-Yu Huang, Mi-Hsueh Tai, Yu-Mei Lin, Chih-Hui Yang, Jei-Fu Shaw

**Affiliations:** 1 Institute of Biotechnology and Chemical Engineering, I-Shou University, Kaohsiung, Taiwan; 2 Department of Biological Science and Technology, I-Shou University, Kaohsiung, Taiwan; 3 Pharmacy Department of E-Da Hospital, Kaohsiung, Taiwan; 4 The School of Chinese Medicine for Post-Baccalaureate, I-Shou University, Kaohsiung, Taiwan; 5 Taiwan Instrument Research Institute, National Applied Research Laboratories, Hsinchu, Taiwan; Tallinn University of Technology, ESTONIA

## Abstract

The purity of chlorophylls plays one of the key role for the production of chlorophyllides. We have designed a facile method for chlorophyll purification by twice solvent extraction. Twice extraction causes the loss of chlorophylls, but the purity of total chlorophylls can be enhanced 182%. Then, the purified chlorophylls can be converted to relatively pure chlorophyllides facilely. The results show that higher purity of chlorophyllides could be obtained when purified chlorophylls (ethanol-hexane extract) was used as starting materials than that of crude chlorophylls (ethanol-only extract). In biocompatibility test, the results showed that the prepared chlorophyllides can be applied as biomaterials. When the prepared chlorophyllides were applied to anticancer tests, they were active both in MCF7 and MDA-MB-231 (multidrug resistant breast cancer cells) cell lines. In addition, the results suggested that the prepared chlorophyllides could be a potential candidate of combination therapy with doxorubicin to breast cancers.

## Introduction

Chlorophyllides were first named by Willstätter and Stoll in 1911 [1]. Chlorophyllides are a phytol-free form of chlorophylls. Chlorophyllides exist in cyanobacteria, green algae and plants, while bacteriochlorophyllides exist in phototrophic bacteria [2]. Chlorophyllides in the photosystems of plants are of two types. Chlorophyllide *a* and chlorophyllide *b* vary slightly in the chemical structure of the porphyrin ring, having molecular formulae of C_35_H_34_MgN_4_O_5_ and C_35_H_32_MgN_4_O_6_, respectively. Chlorophyllides have been used for diverse applications. For example, the antigrowth ability of *Staphylococcus aureus* [3], photodynamic activity as photosensitizers in photodynamic inactivation of *Staphylococcus aureus* [4], antimicrobial activity against *Escherichia coli*, *Serratia marcescens*, *Bacillus thuringiensis* and *Saccharomyces cerevisiae* [5], photoinduced electroreduction in electrodes [6], synthesis of chlorophyll [7] and glutamate [8], and as a sensor for lipid bilayers [9].

Chlorophyllides are unstable under normal conditions. Therefore, considerable research has been devoted to the synthesis of chlorophyllides. The major methods for chlorophyllides preparation are conversion of the protochlorophyllide by enzymes, separation from natural products, and hydrolysis from chlorophyll by enzymes. Several studies have shown that chlorophyllides can be interconverted from protochlorophyllide by protochlorophyllide reductase [10]. In addition, 1,7-phenanthroline and carotenoids have stimulating effects on the conversion of protochlorophyllide into chlorophyllide [11]. Nitric oxide and protochlorophyllide oxidoreductase have inhibitory effects on the conversion of protochlorophyllide to chlorophyllide [12]. Chlorophyllides can be obtained by separation or extraction with different solvents form natural products. Previous researchers have employed methanol [13], acetone/dioxane [14], *N*, *N’*-dimethylformamide [15], diethyl ether/ethanol [16], or hexane [17] to extract chlorophyllides. During the isolation of chlorophyllides, it is important to maintain alkaline conditions, which ensures the retention of the central magnesium ion and prevents the disruption of the tetrapyrrole macrocycle [18]. Many studies have revealed that chlorophyllides can be produced using chlorophyllase. For example, Hsu *et al*. dephytylated chlorophylls to produce chlorophyllides using chlorophyllase isolated from the leaves of *Ficus macrocarpa* [19]. We have expressed chlorophyllases from *Chlamydomonas reinhardtii* and *Brassica oleracea* for the production of chlorophyllides [20, 21].

Previous studies into the synthesis and extraction of chlorophyllides are well established, however, the aforementioned methods have limitations in purity, normality, or stability. For example, our previous study could manufacture crude chlorophyllides, but their impurities limit their applications [22]. In the present study, we have designed a facile method for the manufacture of chlorophyllides using two strategies. Double solvent extraction was employed for the purification of chlorophylls. Second, the purified chlorophylls can then be easily converted to relatively pure chlorophyllides. The prepared chlorophyllides were shown to be potentially useful for combination therapy with doxorubicin, using tests on multidrug resistant breast cancer cell lines.

## Materials and methods

### Materials

Acetone, diethyl ether, ethanol, ethyl acetate, and petroleum ether were purchased from Echo Chemical Co., Ltd. (Taiwan). *n*-hexane, *n*-butanol, and methanol were purchased from Seedchem Company Pty. Ltd. (Melbourne, Australia), Acros Organics (Geel, Belgium), and Aencore Chemical Pty. Ltd. (Surrey Hills, Australia), respectively. Doxorubicin, 3-(4,5-dimethylthiazol-2-yl)-2,5-diphenyl tetrazolium bromide (MTT), potassium hydroxide, sodium phosphate, Triton™ X-100, and chlorophyll a/b standards were purchased from Sigma-Aldrich, Inc. (St. Louis, USA). Sweet potato leaves were purchased from a local market in Kaohsiung, Taiwan. Fibroblast cells (NIH/3T3) and human breast cancer cell lines (MCF7 and MDA-MB-231) were purchased from Bioresource Collection and Research Center (Food Industry Research and Development Institute, Taiwan). Dulbecco’s modified Eagle’s medium (DMEM) and fetal bovine serum (FBS) were obtained from Invitrogen (Carlsbad, USA).

### Solvents extraction for crude chlorophylls

Chlorophyll was extracted using a method based on our previous studies [22, 23]. All procedures were performed in the dark. Fresh leaf samples were washed with water and blotted. 10 g of fresh, clean leaves was weighted and ground into powders using a mortar and pestle, with liquid nitrogen (50 mL) in the dark. Chlorophylls could be extracted by immersing 1 g of leaf powder in 125 mL of petroleum ether, diethyl ether, *n*-hexane, ethyl acetate, acetone, *n*-butanol, ethanol and methanol. After 48 h, each extract was centrifuged at 1500 ×*g* for 5 min, and the chlorophylls extracts were obtained.

### Double extraction of purified chlorophylls

The ethanol extract of the chlorophylls was sequentially extracted using *n-*hexane in the dark. The double extract of chlorophylls was centrifuged at 1500 ×*g* for 5 min, and purified chlorophylls from ethanol-hexane extracts were obtained.

### Measurement of chlorophylls

To measure the concentrations of chlorophyll *a*/*b*, the extracts were passed through a 0.22-μm filter, and the filtrates were analyzed using UV-Vis spectrophotometer (BioTek Instruments, Inc. Winooski, USA). The absorbance was measured at 649 and 665 nm, which are the major absorption peaks of chlorophyll *a* and *b*, respectively. The chlorophyll *a*/*b* contents of the extracts were calculated using previously reported equations [22].

### Preparation of chlorophyllides

Recombinant chlorophyllase was produced using a method based on our previous studies [20]. All procedures were performed in the dark. The reaction mixture contained 0.5 mg of recombinant chlorophyllase, 650 μL of the reaction buffer (contained 100 mM pH 7.4 sodium phosphate and 0.24% Triton™ X-100), and 0.1 mL of 100 mM of chlorophylls extracts. The reaction mixture was incubated at 37°C for 30 min in a shaking water bath, and then the enzymatic reaction was stopped by adding 1 mL of 10 mM KOH. The mixtures were centrifuged at 1500 ×*g* for 10 min, and then the organic and aqueous phases were obtained. After the organic phase was removed, the chlorophyllides could be obtained in the aqueous phase. The solvents of chlorophyllides were removed by evaporation under reduced pressure at 40°C on a rotary evaporator (IKA-Werke, Germany). The concentrated chlorophyllides were processed by lyophilization, weighed, and stored at -80°C for further experiments. Chlorophyllides from ethanol-hexane extracts (purified chlorophylls) were named as purified chlorophyllides.

### Measurement of chlorophyllides

The identification of chlorophyllides was performed by ultra-high-performance liquid chromatography-quadrupole time-of-flight mass spectrometry (UPLC-Q-TOF-MS/MS, Waters Co., Massachusetts, USA) [24]. Before the analysis, the sample solution was prepared by diluting it in 90% acetone. The solution was then centrifuged at 15000 ×*g* for 10 min at 4°C. 3 uL of supernatant was used for analysis. UPLC elution conditions: The elution solvent system comprised 5 mM ammonium formate (A) and acetonitrile (B) at a flow rate of 0.3 mL/min. Samples were eluted using a linear gradient condition: start, 95% A; 2 min, 95% A; 2.5 min, 0 A; 5 min, 40% A; 8 min, 20% A; 10 min, 20% A; 10.5 min, 5% A; 15.5 min, 5% A; 25.5 min, zero A; 30.5 min, zero A; 31 min, 95% A; 33 min, 95% A. Mass spectrometric analyses were performed on a Waters VION LC Q-TOF equipped with an electrospray ionization (ESI) source in positive ion mode. Mass spectrometry conditions: the scanning range was *m/z* 50–1000. The capillary voltage was 1.5 kV, the low collision energy was 6 eV, cone voltage was 20 V, and the higher collision energy was 20–60 eV. The desolvation gas temperature was 500°C. The desolvation gas flow rate was 1000 L/h. Leucine was the lock mass, which was used to correct the mass error. Spectral data analysis and quantification were performed using Unifi software (Version 1.8.2, Waters, UK). The chlorophyllides and pheophorbide *a* standards were purchased from DHI lab products (Hørsholm, Denmark).

### Cell cultures

NIH/3T3, MCF7 and MDA-MB-231 cells were cultured in DMEM supplemented with 10% FBS. The cells were maintained at 37°C under a humidified atmosphere of 5% CO_2_.

### Cell viability test using MTT assay

Cell viability was examined by MTT assay following a previously described procedure [22]. Cells (5 × 10^4^ cells/well) were stimulated with different doses of chlorophyllides (50, 80, 100, 150, and 200 μg/mL). After treatment for 24 h, supernatants were removed from the wells and then 1% of 20 μL MTT solution was added to each well. The plates were incubated for 4 h at 37°C and the optical density was determined at 595 nm using a multi-well Multiskan spectrophotometer (Thermo Fisher Scientific, MA). All measurements made in the 96-well plates were performed using five technical replicates. Further statistical analysis was performed by one way ANOVA using SigmaPlot Version 14.0.

### Combination index assessment

The combination effects of doxorubicin and chlorophyllides at different concentrations were analyzed. The combination index (CI) was determined using ComboSyn software (ComboSyn, Paramus, NJ, USA) where a CI < 1 implied synergism, a CI = 1 implied additive interaction, and a CI > 1 indicated antagonism [25].

## Results and discussions

### Optimization of the method for chlorophyll extraction

Previous researchers have reported that solvents and their polarity determine the profile of the compounds extracted from plants [26–29]. Extracts of various species of plant leaves—using solvents such as ethanol, *n*-hexane, ethyl acetate, dichloromethane, butanol, methanol, petroleum ether, chloroform, and water exhibited anticancer activities [30]. In this study, eight solvents were used to extract chlorophylls from the leaves of sweet potato. **Table 1** shows that neither petroleum ether nor *n*-hexane could easily extract chlorophylls. The extraction yields of chlorophylls in different solvents in descending order were as follows: ethanol > methanol > acetone > ethyl acetate > *n*-butanol > diethyl ether. The ethanol solvent showed the highest extraction yield of both chlorophyll *a* and chlorophyll *b*. The chlorophyll *a* extraction yield of ethanol was 93% higher than that of diethyl ether. The chlorophyll *b* extraction yield of ethanol was 143% higher than that of diethyl ether. We concluded that ethanol was the optimal solvent for chlorophyll extraction. Similar results were reported by Suzuki *et al*. [14] and Al-Alwani *et al*. [31]. Generally, ethanol extraction was not able to exclude hydrophilic compounds, such as anthocyanin and flavonoids, indicating that these components may be co-extracted with chlorophylls by ethanol [32]. In this study, the chlorophylls from ethanol extract were called crude chlorophylls extracts.

**Table 1:** Contents of chlorophyll *a* and chlorophyll *b* in eight extracts obtained using various solvents.

Solvent	Yield (mg/gDw)	
	Chlorophyll *a*	Chlorophyll *b*
Petroleum ether	N/A	N/A
*n*-Hexane	N/A	N/A
Diethyl ether	20.5	7.1
*n*-Butanol	24.9	7.5
Ethyl acetate	27.3	13.6
Acetone	31.6	14.2
Methanol	37.4	16.3
Ethanol	39.6	17.3

gDW: gram dry weight

N/A: not available

To exclude the hydrophilic compounds and obtain purer chlorophylls, a second step solvent extraction was employed. We propose that the additional hexane extraction could removes the undesired water-soluble compounds from the crude chlorophylls extract. The chlorophyll *a* extraction yield from double solvent extraction (*e*.*g*. ethanol-hexane extraction) was around 9.8% lower than that of ethanol-only extraction. The chlorophyll *b* extraction yield from ethanol-hexane extraction was 33.41% lower than that from ethanol-only extraction. The total chlorophylls from ethanol-hexane extraction were decreased 17.03% compared with ethanol-only extraction. Double extraction caused the loss of both chlorophyll *a* and chlorophyll *b*, but the purity of chlorophyll *a* and chlorophyll *b* was enhanced by 146.56% and 35.49%, respectively.

### Preparation of chlorophyllides from crude or purified chlorophylls

**Fig 1:**
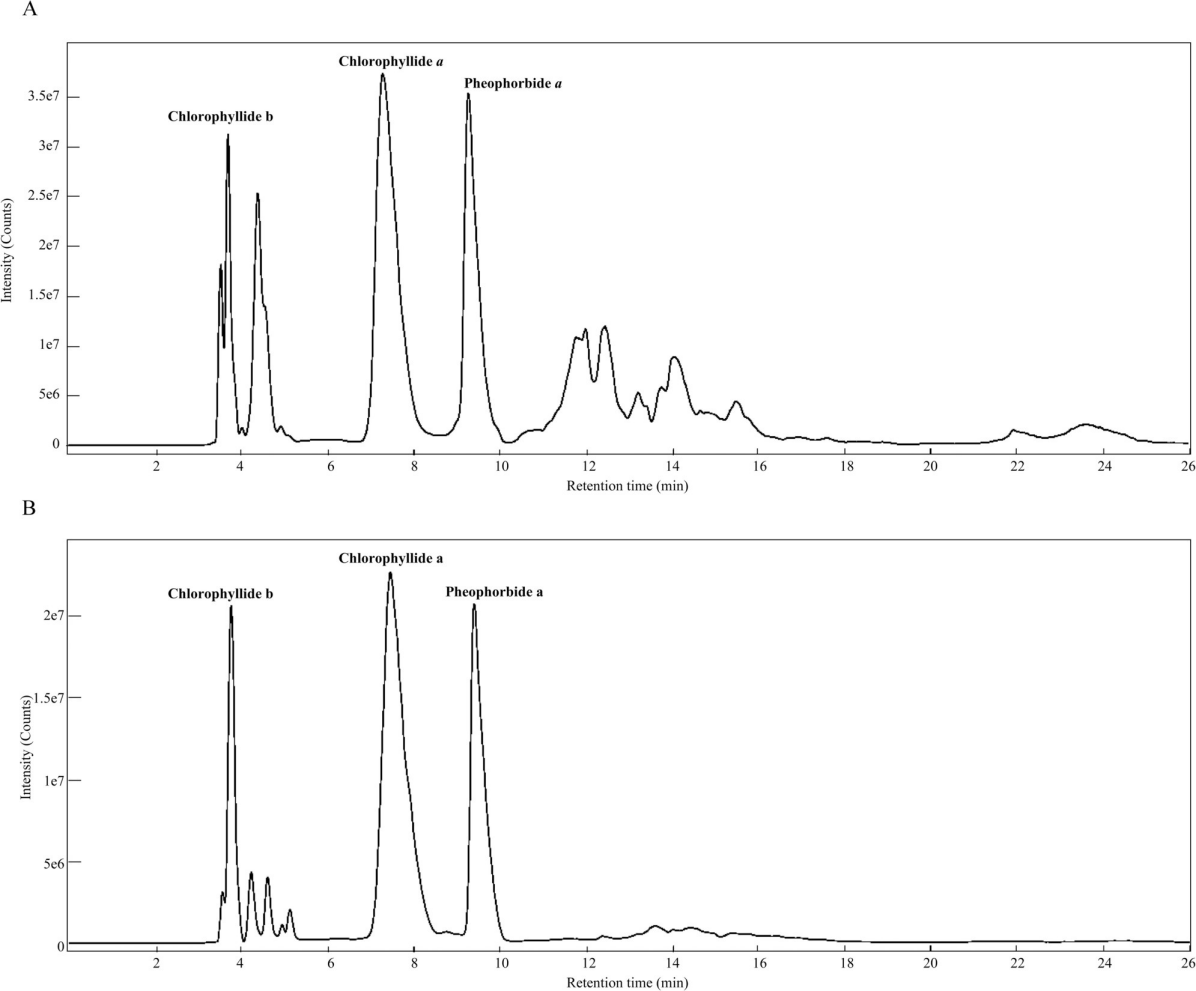
The UPLC-Q-TOF/MS data of (A) crude chlorophyllides and (B) purified chlorophyllides.

**Fig 2:**
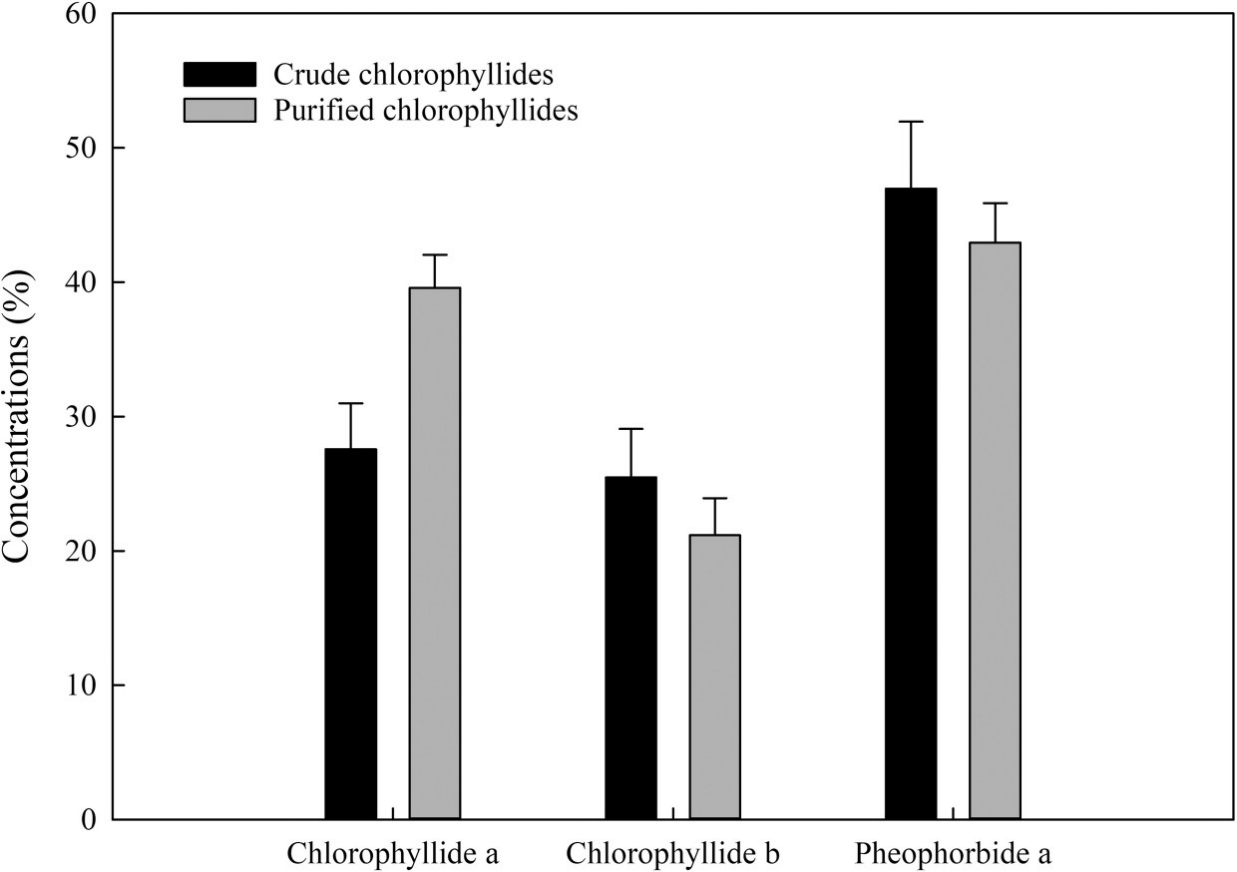
Comparison of major compounds from crude chlorophyllides and purified chlorophyllides. Data representing mean ±SE were obtained from three and five independent replicates of crude chlorophyllides and purified chlorophyllides, respectively. Further statistical analysis was performed by one way ANOVA using SigmaPlot Version 14.0. The statistical result indicated that there is not a statistically significant difference.

Chlorophyllides can be prepared from crude chlorophylls (ethanol-only extract) or purified chlorophylls (ethanol-hexane extract). After chlorophyll was hydrolyzed by recombinant chlorophyllase, the chlorophyllides could be obtain in the aqueous phase. UPLC-Q-TOF/MS was used to analyze the purity of the chlorophyllides (**Fig 1**). The products were different when two different starting materials were used (**Fig 2**). When crude chlorophylls (ethanol-only extract) were used as starting materials, at least ten peaks of UPLC were present, as shown in **Fig 1A**. When purified chlorophylls (ethanol-hexane extract) were used as starting materials, three main peaks could be detected, as shown in **Fig 1B**. Compared to standards, peaks 1, 2, and 3 were referred to chlorophyllide *b*, chlorophyllide *a* and pheophorbide *a*, respectively [33]. **Fig 2** shows the comparison of the three hydrolyzed products from two type of starting materials. The results showed that higher chlorophyllide *a* (143.5%) was obtained when purified chlorophylls (ethanol-hexane extract) were used as starting materials than the crude chlorophylls (ethanol-only extract). Lower chlorophyllide *b* (16.8%) was obtained from purified chlorophylls. Moreover, the total chlorophyllides from purified chlorophylls were increased about 14.55%. A trend toward increased level was observed in both purified chlorophylls and purified chlorophyllides. Therefore, purified chlorophylls were therefore a better starting material for the manufacture of chlorophyllides.

### Biocompatibility of chlorophyllides

**Fig 3:**
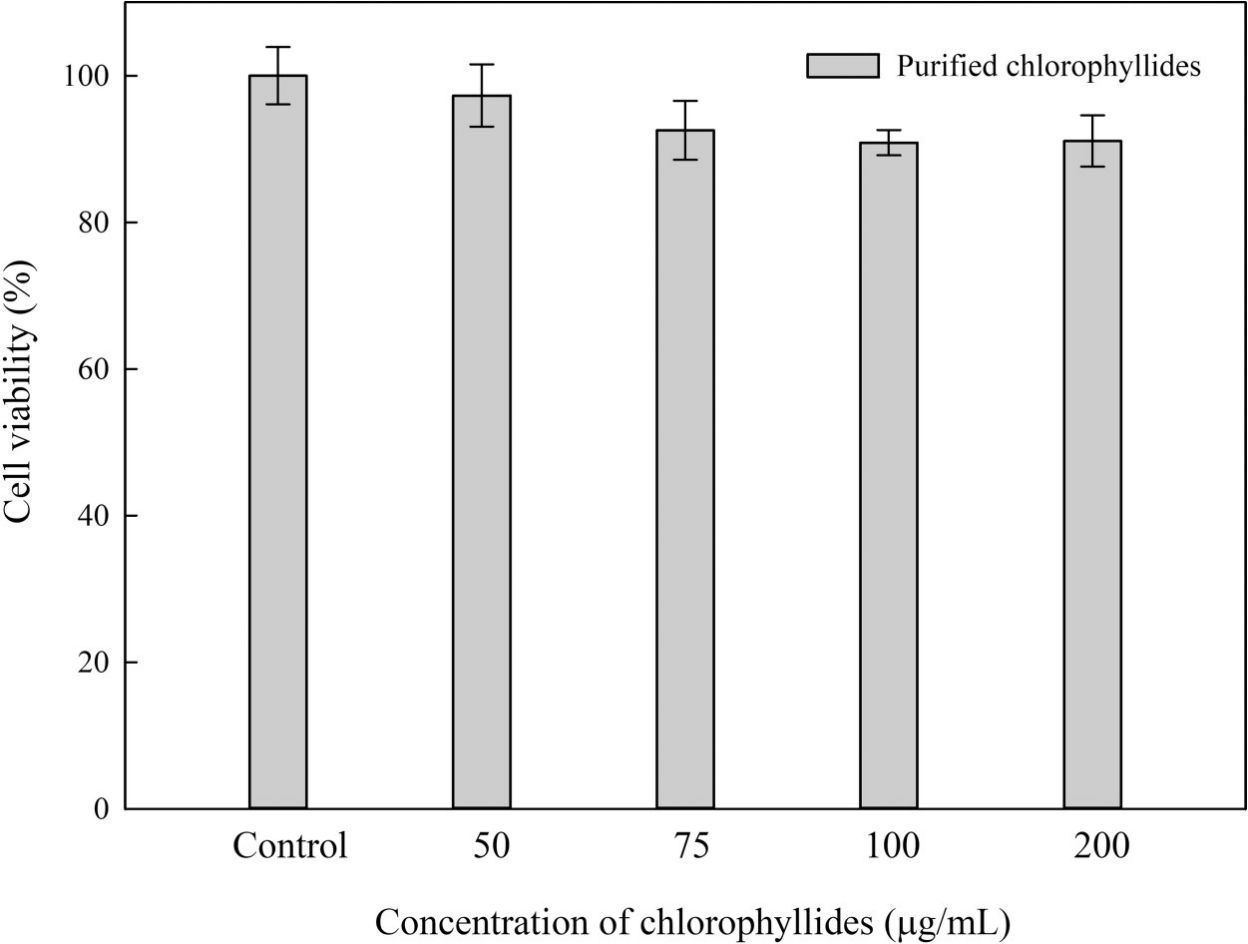
MTT assay of chlorophyllides in NIH/3T3 cells. The results showed that when the concentration was 200 μg/mL, there is no statistical difference between the control group (chlorophyllides absent). Data representing mean ±SE from five independent replicates was subjected to further statistical analysis by one way ANOVA using SigmaPlot Version 14.0. One-way ANOVA for this experiment returns *p* = 0.511.

Cytotoxic effects could provide useful and crucial information about materials used in biomedical research. The purifed chlorophyllides were obtained from ethanol-hexane-extracted chlorophylls. To increase the applicability of the purified chlorophyllides as a biomaterial, we studied their biocompatibility using MTT assays (**Fig 3**). NIH/3T3 cells were cultured with the purifed chlorophyllides (0–200 μg/mL) for 24 h. The cell viability of purified chlorophyllides was approximately 90%–95%. There is no statistical difference between the control group (chlorophyllides absent). Therefore, the purified chlorophyllides could be used as biomaterials.

### Cytotoxic effects of chlorophyllides in breast cancer cell lines

**Fig 4:**
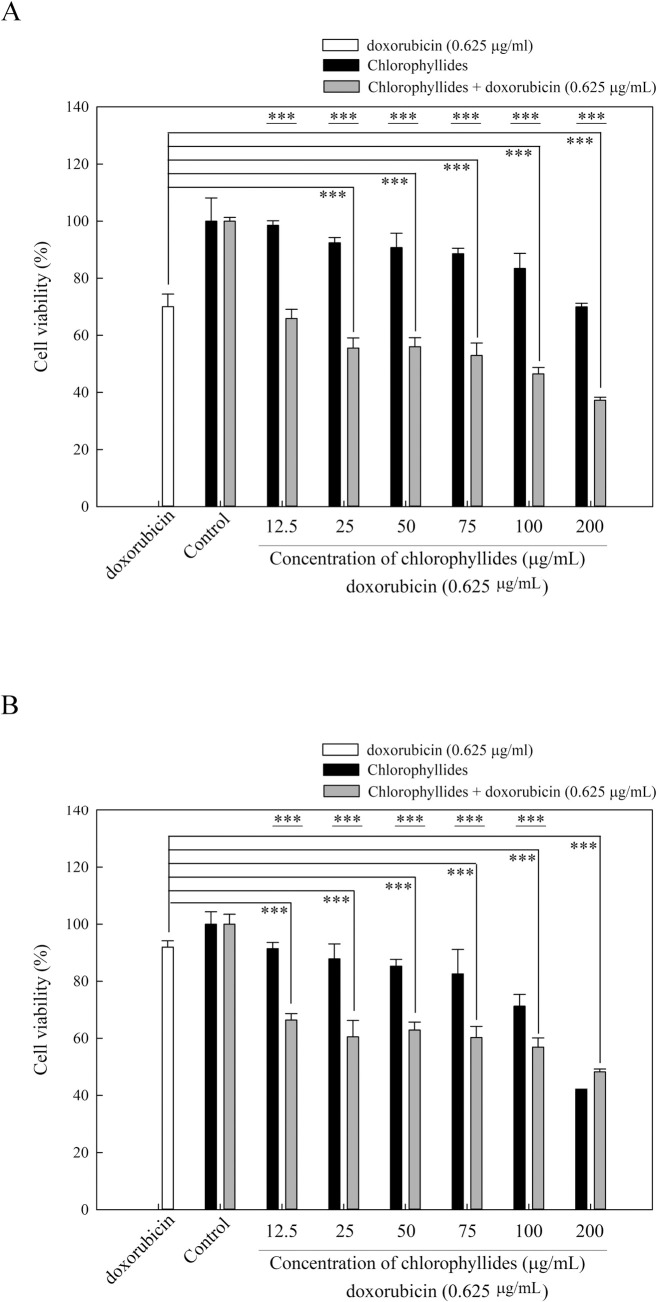
MTT assay of chlorophyllides and the combination of doxorubicin (0.625 μg/mL) and chlorophyllides in MCF7 cell lines and MDA-MB-231 cell lines. Data representing mean ±SE from five independent replicates was subjected to further statistical analysis by one way ANOVA using SigmaPlot Version 14.0. *p* < 0.001 ***. A. MCF7 cells. B. MDA-MB-231 cells.

The purified chlorophyllides were used to test the cytotoxic effects in breast cancer cell lines. Breast cancer is the second most common cause of cancer-related mortality in women [34]. One of the major challenges in breast cancer treatment is multiple drug resistance, which may cause cross resistance to chemotherapeutics [35]. MCF7 cell lines are epithelial luminal breast cell lines, and are widely used for breast cancer studies. MDA-MB-231 cells are one type of multidrug-resistant breast cancer cells, that possess an intermediate response to chemotherapy [36]. In this study, both MCF7 and MDA-MB-231 cell lines were used in the MTT assay (**Fig 4**). In **Fig 4A**, the chlorophyllides are shown to be active in MCF7 cell lines. The cell viabilities of chlorophyllides in MCF7 cell lines were decreased in a dose-dependent manner (50–200 μg/mL).

To further examine the cytotoxic effects of the purified chlorophyllides in breast cancer with multiple drug resistance, MDA-MB-231 cell lines were used. The purified chlorophyllides significantly decreased the viability of MDA-MB-231 cell lines, as shown in **Fig 4B**. Cell viabilities of the chlorophyllides exhibited strong dose-response curves. For example, the cell viabilities with chlorophyllides at 200 μg/mL were 47.56%, indicating that the purified chlorophyllides could effectively inhibit the cell growth of MDA-MB-231. These results suggest that the MDA-MB-231 cell lines were more sensitive to chlorophyllides than the MCF7 cell lines. In addition, IC_50_ of 188.91 μg/mL was obtained using the purified chlorophyllides in MDA-MB-231 cell lines.

### Combination therapy in breast cancer cell lines

Doxorubicin is currently one of the most effective chemotherapeutic drugs for breast cancer treatment [37]. However, doxorubicin resistance in breast cancer cell lines has serious side effects [38]. In the search for a more effective chemotherapeutic method, combination therapy with compounds from natural products is a promising and alternative method for treating multidrug-resistant cancers [39]. For example, tanshinone IIA could enhance the chemosensitivity of breast cancer cells to doxorubicin by downregulating the expression of MDR-related ABC transporters [39]. Toosendanin, ursolic acid, wogonin, curcuminoid derivatives, flavonoids, sulforaphane, and others could successfully enhance the chemosensitivity of breast cancer cells. In this study, the purified chlorophyllides were shown to produce cytotoxic effects in MDA-MB-231 cell lines. Combination therapy using doxorubicin and the prepared chlorophyllides was studied to identify synergistic or antagonistic effects. In the MDA-MB-231 cell lines, cytotoxicity effects were increased in a dose-dependent manner using the combination therapy, as shown in **Fig 4B**. The cell viabilities resulting from the combination therapy with 0.625 μg/mL doxorubicin and chlorophyllides (0–200 μg/mL) were 49–65%. The results demonstrated that chlorophyllides could enhance the chemosensitivity of breast cancer cells to doxorubicin.

Drug–drug combinations are convenient models of additivity and provide valuable insights into the significance of synergistic or antagonistic interactions [40]. To ascertain whether chlorophyllides influence the cytotoxic effects of doxorubicin, various concentrations of doxorubicin and chlorophyllides were selected for combination cytotoxicity (**Table 2**). Combinations of chlorophyllides from 12.5 to 100 μg/mL and doxorubicin at 0.625 μg/mL exhibited a CI of 0.07109–0.60723, thus showing synergistic interactions in MDA-MB-231 cells. The best synergism rate (CI = 0.07109) was observed with doxorubicin at a dose of 0.625 μg/mL combined with purified chlorophyllide at dose of 12.5 μg/mL. Using chlorophyllides at dose of 200 μg/mL with doxorubicin at 0.625 μg/mL, the CI was above 1, indicating antagonism. Combinations of chlorophyllides at 100 μg/mL and doxorubicin from 0.625 to 20 μg/mL produced a CI of 0.24743–0.49972, thus showing synergistic interactions. The results indicate that the synergistic effects were stronger when the doxorubicin and chlorophyllides were combined at relatively low concentrations. The dose of chlorophyllides at 200 μg/mL with doxorubicin at 0.625 μg/mL suggests that combination with chlorophyllides could decrease the dosage of doxorubicin. The detailed mechanism of chlorophyllides in combination therapy with doxorubicin in breast cancer will be studied in the future. We therefore demonstrated that purified chlorophyllides could be a potential candidate for combination therapy to breast cancers with multiple drug resistance.

**Table 2:** Combination effects of doxorubicin and chlorophyllides in the MDA-MB-231 cell line.

Doxorubicin (μg/mL)	Chlorophyllides (μg/mL)	Combination index (CI)
0.625	12.5	0.07109
0.625	25	0.13802
0.625	50	0.29149
0.625	75	0.43179
0.625	100	0.60723
0.625	200	1.07026
1.25	100	0.49972
2.5	100	0.44011
5	100	0.39904
10	100	0.35991
20	100	0.24743

## Conclusions

A new method for the production of chlorophyllides using double extractions was successfully developed. For the medicinal applications of chlorophyllides, ethanol and hexane are generally recognized as safe solvents for use. Purified chlorophyllides were obtained in the aqueous phase when the purified chlorophylls were hydrolyzed with chlorophyllase. The results revealed that the purity of chlorophyllide *a* and chlorophyllides increased by 43.5% and 14.55%, respectively. The MTT assays, the purified chlorophyllides were effective against both MCF7 and MDA-MB-231 cells. In addition, the results illustrated that the combination of chlorophyllides and doxorubicin showed synergistic cytotoxic effects. We have demonstrated that purified chlorophyllides could be a potential candidate for combination therapy in multiple drug resistant breast cancers.

## Supporting information

S1 Fig MTT assay of (A) chlorophyllides in MCF7 cell lines, (B) chlorophyllides in MDA-MB-231 cell lines, (C) the combination of doxorubicin (0.625 μg/mL) and chlorophyllides in the MDA-MB-231 cell lines. The statistical method used in this article is one way ANOVA using SigmaPlot Version 14.0.
(DOCX)

S1 Table MS data from the purified chlorophyllides. (DOCX)

S1 Graph abstract (TIF)
