# Supplementary material for: Facile production of chlorophyllides using recombinant CrCLH1 and their cytotoxicity towards multidrug resistant breast cancer cell lines
Source: PLoS One. 2021 Apr 30;16(4):e0250565. doi: 10.1371/journal.pone.0250565 (PMC8087012; doi:10.1371/journal.pone.0250565)
Supplement: S1 Fig — MTT assay of (A) chlorophyllides in MCF7 cell lines, (B) chlorophyllides in MDA-MB-231 cell lines, (C) the combination of doxorubicin (0.625 μg/mL) and chlorophyllides in the MDA-MB-231 cell lines. The statistical method used in this article is one way ANOVA using SigmaPlot Version 14.0. (DOCX) [file pone.0250565.s001.docx]

A


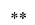

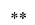

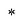


B


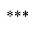

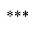

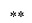

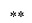

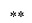


C


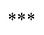

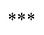

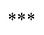

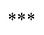

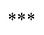

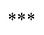


**S1 Fig.** MTT assay of (A) chlorophyllides in MCF7 cell lines, (B) chlorophyllides in MDA-MB-231 cell lines, (C) the combination of doxorubicin (0.625 μg/mL) and chlorophyllides in the MDA-MB-231 cell lines. The statistical method used in this article is one way ANOVA using SigmaPlot Version 14.0.
