# Supplementary material for: Facile production of chlorophyllides using recombinant CrCLH1 and their cytotoxicity towards multidrug resistant breast cancer cell lines
Source: PLoS One. 2021 Apr 30;16(4):e0250565. doi: 10.1371/journal.pone.0250565 (PMC8087012; doi:10.1371/journal.pone.0250565)
Supplement: S1 Table — (DOCX) [file pone.0250565.s002.docx]

**S1 Table.** MS data from the purified chlorophyllides

|  | Identification | Rt (min) | Adducts | Molecular formula | Neutral Mass (kDa) | Counts |
| --- | --- | --- | --- | --- | --- | --- |
| 1 | Chlorophyllide *b* | 3.63 | +H | C_35_H_32_MgN_4_O_6_ | 628.21723 | 108,929,783 |
| 2 | Chlorophyllide *a* | 3.8 | +H | C_35_H_34_MgN_4_O_5_ | 614.23796 | 111,749,467 |
| 3 | Pheophorbide *a* | 7.37 | +H, +Na | C_35_H_36_N_4_O_5_ | 592.26857 | 800,915,383 |
