# Supplementary figures and images for: Facile production of chlorophyllides using recombinant CrCLH1 and their cytotoxicity towards multidrug resistant breast cancer cell lines
Source: PLoS One. 2021 Apr 30;16(4):e0250565. doi: 10.1371/journal.pone.0250565 (PMC8087012; doi:10.1371/journal.pone.0250565)

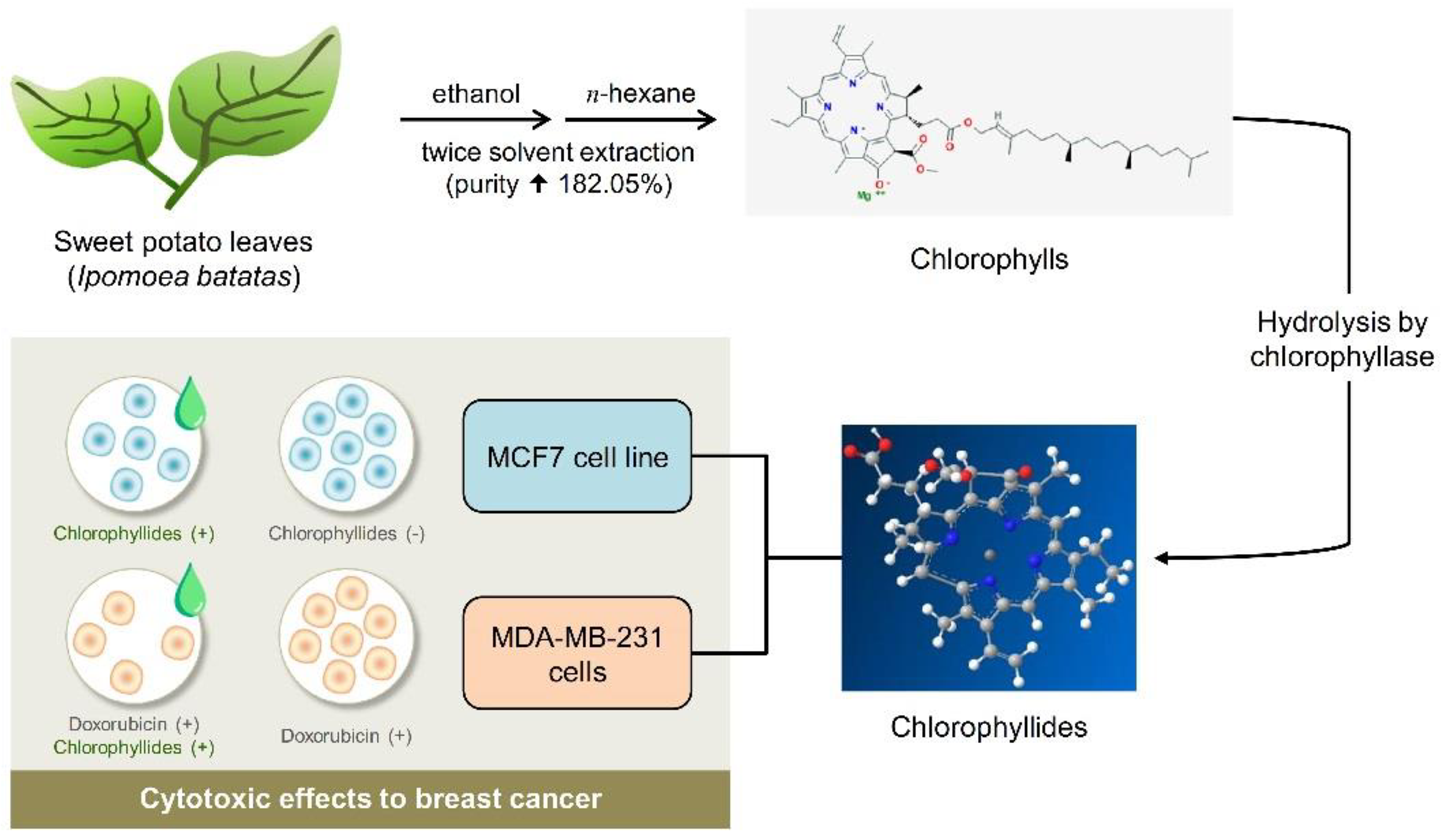

Supplement: S1 Graph abstract — (TIF) [file pone.0250565.s003.tif]
